# Interrelationships of frailty, hemoglobin, cognition, and depressive symptoms in aging: a path analysis of the ELSI-Brazil study

**DOI:** 10.1590/0102-311XEN105124

**Published:** 2025-04-28

**Authors:** Ligiana Pires Corona, Gabriela Benatti de Oliveira, Lara Vilar Fernandes, Natalie Bitencourt Ramos, Carolina Neves Freiria, Luciana Scarlazzari Costa

**Affiliations:** 1 Faculdade de Ciências Aplicadas, Universidade Estadual de Campinas, Limeira, Brasil.; 2 Universidade São Francisco, Bragança Paulista, Brasil.

**Keywords:** Frailty, Anemia, Depression, Structural Equation Modeling, Fragilidad, Anemia, Depresión, Modelado de Ecuaciones Estructurales

## Abstract

This study analyzed the interrelationships of anemia, depression, and cognition, as well as some of their associated factors to understand the paths to frailty. Data from 2,174 baseline participants of the *Brazilian Longitudinal Study of Aging* (ELSI-Brazil) were used. Path analysis was used to assess the relationships of exogenous variables (place of residence, education level, number of diseases, diet score, and number of natural teeth), one endogenous variable (frailty), and three mediators (cognition, depression, and hemoglobin level). Cognition and hemoglobin level showed a negative path to frailty, while depressive symptoms showed a positive path. Among the exogenous variables, rural area had a negative effect on hemoglobin, depressive symptoms, and frailty; a higher education level showed a positive path to cognition; number of diseases showed a negative path to hemoglobin and a positive path to depressive symptoms and frailty; diet score showed a negative path to hemoglobin and depressive symptoms; number of teeth had a positive effect on cognition and hemoglobin and a negative effect on frailty. Two paths without direct effects had significant indirect effects - rural area showed an indirect relationship with cognition via hemoglobin and depressive symptoms; and education level showed an indirect path to frailty, mediated by the three intermediate variables. These results show complex interrelationships of frailty, hemoglobin, cognition, and depressive symptoms, which help understand the syndrome in a broad way and support the planning of more comprehensive prevention and intervention measures.

## Abstract (es)

Este estudio tuvo como objetivo evaluar las interrelaciones entre la anemia, la depresión y la cognición, así como algunos de sus factores asociados, para comprender las trayectorias hacia la fragilidad. Se utilizaron datos de 2.174 participantes de la línea de base del *Estudio Longitudinal sobre la Salud de Ancianos Brasileños* (ELSI-Brasil). El análisis de la trayectoria se utilizó para evaluar las relaciones entre las variables exógenas (lugar de residencia, nivel de estudios, número de enfermedades, puntuación de alimentación y número de dientes naturales), una endógena (fragilidad) y tres mediadores (cognición, depresión y nivel de hemoglobina). Los análisis mostraron que la cognición y la hemoglobina tuvieron una trayectoria negativa para fragilidad; y que los síntomas depresivos presentaron una trayectoria positiva. Entre las variables exógenas, las áreas rurales tuvieron un efecto negativo sobre la hemoglobina, los síntomas depresivos y la fragilidad; mayor nivel de estudios tuvo una trayectoria positiva para la cognición; el número de enfermedades tuvo una trayectoria negativa para la hemoglobina, y positiva para los síntomas depresivos y la fragilidad; la puntuación de alimentos tuvo una trayectoria negativa para la hemoglobina y los síntomas depresivos; el número de dientes tuvo un efecto positivo sobre la cognición y la hemoglobina; y negativo sobre la fragilidad. Dos trayectorias sin efecto directo tuvieron efectos indirectos significativos: las áreas rurales mostraron una relación indirecta con la cognición a través de la hemoglobina y los síntomas depresivos; y el nivel de estudios tuvo una trayectoria indirecta para fragilidad mediada por las tres variables intermedias. Estos resultados muestran interrelaciones complejas entre la fragilidad, la hemoglobina, la cognición y los síntomas depresivos, elementos importantes para comprender el síndrome de una manera amplia, de modo que se puedan promover medidas de prevención e intervención más integrales.

## Introduction

Geriatric syndromes, such as frailty, are of increasing concern to health professionals due to the exponential aging of the population in recent decades. The prevalence of frailty varies according to the diagnostic criteria used ^1^. In Brazil, a prevalence of 8.1% was found among individuals aged 60-69 years and 20.9% among individuals aged 70 years and older ^2^. It increases the risk of functional and cognitive decline, hospitalization, morbidity and mortality, leading to poorer quality of life, reduced longevity, and increased health care costs.

The pathophysiology of frailty is characterized by an imbalance in homeostasis, creating a pathological cycle in a negative spiral of functional decline, as proposed by Fried et al. ^3^, with clinical signs of reduced strength, fatigue, slowness, low physical activity, and weight loss; it is considered a complex and interconnected phenomenon ^4^.

Frailty has been associated with anemia ^5^ ^,^ ^6^, cognition ^7^ ^,^ ^8^, depression ^9^ ^,^ ^10^ ^,^ ^11^, diet ^12^ ^,^ ^13^, lifestyle ^14,15^, chronic noncommunicable diseases (CNCDs) ^16^, and sarcopenia ^17^, but to date, there is limited evidence of the complex relationships between these conditions and the clinical manifestation of frailty.

Understanding this complexity can contribute to the development of broader prevention and treatment actions for frailty and support the creation of public policies to improve the quality of life and longevity of the population. Therefore, this study aimed to analyze the interrelationships of anemia, depression, and cognition, as well as some associated factors to evaluate the paths to frailty.

## Methods

This study uses baseline data from the *Brazilian Longitudinal Study of Aging* (ELSI-Brazil, acronym in Portuguese), a household-based survey conducted in 2015-2016 with a representative national sample of the population aged 50 years and older. Methodological details of the study (selection, sample, procedures) are described on the study website (https://elsi.cpqrr.fiocruz.br/) and in previous publications ^18^.

The study was conducted in 70 municipalities of the five major regions of Brazil, with a final sample of 9,412 participants. A subsample was also selected using probability sampling for blood collection, totaling 2,174 individuals. Blood samples were collected at the homes of participants; then they were prepared and sent to the central laboratory for analysis. To ensure sample quality and viability, a best practice protocol was observed, which included packaging with dry ice and temperature monitoring during transport ^19^.

ELSI-Brazil was approved by the Ethics Research Committee of the Oswaldo Cruz Foundation at Minas Gerais State (CAAE 34649814.3.0000.5091), and all interviewees signed an informed consent form to participate in the study ^18^ ^,^ ^19^.

The main outcome variable was frailty, defined as the sum of five criteria presented in previous publications ^2^ ^,^ ^3^ ^,^ ^4^: self-reported unintentional weight loss (last three months), weakness (grip strength in the lower quintile stratified by sex, and body mass index - BMI - quartiles), low gait speed (upper quintile of three-meter walk time, stratified by sex and height), fatigue (responses to two questions from the *Center for Epidemiological Studies Depression Questionnaire* - CES-D) ^20^ ^,^ ^21^; and low level of physical activity (lowest quintile of weekly caloric expenditure calculated using the *Short Form of the International Physical Activity Questionnaire* - IPAQ ^22^, stratified by sex).

Three conditions were selected as possible mediators: serum hemoglobin levels (mg/L), cognition (sum of immediate and delayed memory, and verbal fluency - the higher the score, the better) ^23^ ^,^ ^24^, and number of depressive symptoms, identified by the CES-D depression scale (ranging from 0 to 8 - the higher the score, the higher the number of depressive symptoms) ^21^.

The explanatory variables were: place of residence (urban area, rural area); education (no education, primary, secondary, high school, higher education); number of natural teeth in the mouth (none, 1-9, 10-19, ≥ 20); number of diseases (sum of 13 self-reported CNCDs: hypertension, diabetes, hypercholesterolemia, heart disease, stroke, chronic lung disease, arthritis/rheumatism, osteoporosis, chronic back problems, cancer, chronic kidney failure, Parkinson’s or Alzheimer’s disease); and food groups of adequate consumption (consumption of fruits/juice, vegetables, and meats at least five times a week and consumption of fish at least once a week; sum ranging from 0 to 4 adequate groups).

For descriptive analyses, mean (standard error), minimum and maximum values, and percentages were used. To assess the interrelationships of the variables and the frailty score, a structural equation model and path analysis were used considering five exogenous variables (place of residence, education, number of diseases, diet score, and number of natural teeth), one endogenous variable (frailty), and three mediators, which could be either exogenous or endogenous (cognition, depression, and hemoglobin level). The model adequacy was assessed using the chi-square test (root mean square error of approximation - RMSEA; values < 0.05 indicate excellent fit), standardized root mean square residual (SRMR; acceptable values < 0.08), Tucker-Lewis index (TLI), and comparative fit index (CFI), ranging from 0 to 1; the closer to 1, the better the fit.

Data were analyzed using Stata, version 14.0 (https://www.stata.com), with a significance level of 5%, using sample weights calculated specifically for those who provided a blood sample through the survey package for complex samples.

## Results

Most participants of the ELSI-Brazil study live in urban areas, completed primary school (59.8%), have an average of 2.38 diseases, an average hemoglobin level of 13.71mg/dL, and 1.06 components of frailty (data not shown in the table).

**Figure 1:**
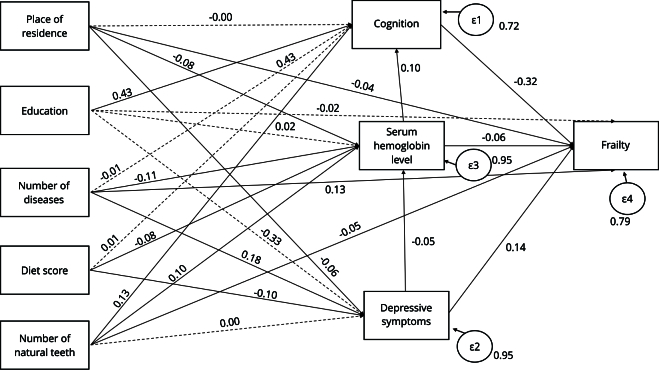
Model of direct relationships of variables in the path analysis.

Note: only solid lines represent significant associations (p < 0.05). Reference categories of qualitative variables - age (50-59 years); place of residence (urban area); education (no education); number of natural teeth (none).

Figure 1 shows the direct effects of the variables. Cognition and hemoglobin showed a negative path to frailty, while depressive symptoms showed a positive path to frailty. Among the exogenous variables, rural area showed a negative effect on hemoglobin, depressive symptoms, and frailty; higher education level showed a positive path to cognition; number of diseases showed a negative path to hemoglobin and a positive path to depressive symptoms and frailty; diet score showed a negative path to hemoglobin and depressive symptoms; and number of teeth had a positive effect on cognition and hemoglobin and a negative effect on frailty.

Table 1 shows the total effects of all path analyses and Table 2 shows data on indirect effects observed in the model.

**Table 1:** Total effects of path analysis.

Total effects	Standardized coefficient	Standard error	p-value
Cognition			
Depressive symptoms	-0.006	0.008	0.016
Serum hemoglobin level	0.109	0.105	< 0.001
Place of residence	-0.012	0.394	0.528
Education	0.437	0.131	< 0.001
Number of diseases	-0.030	0.085	0.113
Diet score	0.009	0.139	0.609
Number of natural teeth	0.143	0.130	< 0.001
Depressive symptoms			
Place of residence	-0.064	0.153	0.003
Education	-0.034	0.051	0.158
Number of diseases	0.187	0.033	< 0.001
Diet score	-0.102	0.054	< 0.001
Number of natural teeth	-0.005	0.050	0.819
Hemoglobin			
Depressive symptoms	-0.059	0.012	0.008
Place of residence	-0.079	0.083	< 0.001
Education	0.024	0.028	0.318
Number of diseases	-0.124	0.018	< 0.001
Diet score	-0.076	0.029	0.001
Number of natural teeth	0.105	0.028	< 0.001
Frailty			
Cognition	-0.320	0.003	< 0.001
Depressive symptoms	0.154	0.009	< 0.001
Serim hemoglobin level	-0.099	0.017	< 0.001
Place of residence	-0.046	0.064	0.034
Education	-0.170	0.021	< 0.001
Number of diseases	0.182	0.014	< 0.001
Diet score	-0.014	0.022	0.512
Number of natural teeth	-0.112	0.021	< 0.001

**Table 2:** Indirect effects of path analysis.

Indirect effects	Mediator	Standardized coefficient	Standard error	p-value
Cognition				
Depressive symptoms	Serum hemoglobin level	-0.006	0.008	0.016
Place of residence	Serum hemoglobin level/Depressive symptoms	-0.008	0.058	0.002
Education	Serum hemoglobin level/Depressive symptoms	0.003	0.017	0.325
Number of diseases	Serum hemoglobin level/Depressive symptoms	-0.013	0.015	< 0.001
Diet score	Serum hemoglobin level/Depressive symptoms	-0.008	0.020	0.004
Number of natural teeth	Serum hemoglobin level/Depressive symptoms	0.011	0.020	0.001
Serum hemoglobin level				
Place of residence	Depressive symptoms	0.004	0.007	0.049
Education	Depressive symptoms	0.002	0.002	0.213
Number of diseases	Depressive symptoms	-0.011	0.004	0.011
Diet score	Depressive symptoms	0.006	0.003	0.021
Number of natural teeth	Depressive symptoms	-0.000	0.001	0.819
Frailty				
Depressive symptoms	Serum hemoglobin level	0.006	0.001	0.021
Serum hemoglobin level	Cognição	-0.035	0.005	< 0.001
Place of residence	Serum hemoglobin level/Depressive symptoms/Cognition	-0.000	0.023	0.950
Education	Serum hemoglobin level/Depressive symptoms/Cognition	-0.147	0.011	< 0.001
Number of diseases	Serum hemoglobin level/Depressive symptoms/Cognition	0.046	0.006	< 0.001
Diet score	Serum hemoglobin level/Depressive symptoms/Cognition	-0.013	0.008	0.089
Number of natural teeth	Serum hemoglobin level/Depressive symptoms/Cognition	-0.053	0.008	< 0.001

Fit adequacy measures by path analysis: CFI (comparative fit index) = 0.999; TLI (Tucker-Lewis index) = 0.970; SRMR (standardized root mean square residual) = 0.005; RMSEA (root mean square error of approximation) = 0.027; chi-squared test for fit quality = 0.000; chi-squared ratio (χ2/g.l.) = 0.114. The total R^2^ of the model was 35%.

Two paths without a direct effect had significant indirect effects: living in a rural area showed an indirect relationship with cognition via hemoglobin and depressive symptoms; and education level showed an indirect path to frailty, mediated by the three intermediate variables. The model proved to be adequate in terms of adjustment parameters.

## Discussion

In this study, we demonstrated interrelationships among frailty, hemoglobin, cognition, and depressive symptoms, and direct and indirect pathways that some of their classic determinants present. To our knowledge, this is the first study to show all these effects in a single analysis using a representative sample of the population aged 50 and over in the country.

Frailty is a geriatric syndrome caused by pathophysiological mechanisms that deregulate multiple systems, depleting reserves and compromising homeostasis in the presence of stressors. It involves systems of energy production, distribution, and use, including hormonal, immunological, inflammatory, and neurological processes. Thus, the cycle of reduced energy supply and demand leads to impaired function within and between systems. In this scenario, anemia is involved in the pathophysiology of the syndrome, due to a reduction in oxygen transport capacity, which can lead to tissue hypoxia ^25^, and consequently sarcopenia, osteoporosis, cardiac dysfunction, and progression of kidney disease ^26^ ^,^ ^27^. Also, anemia can contribute to clinical manifestations of weakness and exercise intolerance ^28^, which are components of frailty.

However, most studies demonstrate the direct effect of already established anemia on frailty syndrome, and not on hemoglobin gradient; besides, these are studies with more local characteristics. In Brazil, the *Study Health, Well-Being and Aging* (SABE study, acronym in Portuguese) showed this relationship in older people from the city of São Paulo only, in which lower hemoglobin levels were associated with a higher number of positive criteria for frailty ^6^.

Lower hemoglobin levels also contribute to lower cognitive capacity and a higher chance of depressive disorders because tissue hypoxia has an important effect on the nervous system, with two possible types of association - of cause or consequence.

Anemia can lead to depression due to deficiency of vitamins such as folate and vitamin B12, which decrease the production of S-adenosylmethionine, or can increase the production of homocysteine. At the same time, fatigue and lack of interest in daily activities (such as shopping, cooking, and others), which are commonly seen in depressed individuals, can affect the quality of diet, favoring the development of anemia; and appetite can also be severely impacted ^29^ ^,^ ^30^ ^,^ ^31^ ^,^ ^32^. A cohort of Chinese individuals showed that the incidence of anemia in 4-year follow-up was higher in individuals with depressive symptoms, and the higher the score on the test, the higher the incidence of anemia ^33^. In this study, the higher the number of depressive symptoms, the lower the circulating hemoglobin concentration, but it was not possible to assess causal associations considering the cross-sectional nature of data.

The same may be observed in cognitive capacity - individuals with established cognitive impairment may find it difficult to buy and prepare foods, feed themselves, and may have poor swallowing and, therefore, poor nutrition, which may trigger nutritional anemia ^34^. However, anemia as a cause of cognitive impairment is the most accepted pathway. Two recent reviews show that anemia increased the incidence of both general cognitive impairment and dementia, possibly due to prolonged tissue hypoxia in brain tissue ^35^ ^,^ ^36^. Depression is also positively associated with physical and cognitive frailty ^37^ ^,^ ^38^ through several physiological mechanisms, with changes in synaptic function, protein transport, and mitochondrial function.

This interrelationship between the various conditions assessed in this study (frailty, low hemoglobin level, depression, and cognition) seems to have in common a condition named inflammaging, which is an inflammatory state related to aging that increases oxidative stress, negatively impacting the systems involved ^39^ ^,^ ^40^. Inflammation may be directly associated with frailty through independent mechanisms ^41^ and changes in the erythropoietic response ^42^; it is considered an important determinant in the development of chronic anemia in older adults ^27^ ^,^ ^42^ ^,^ ^43^. According to Guralnik et al. ^44^, at least one third of anemia cases in aging can be attributed to CNCDs (especially renal diseases) and/or inflammation.

In addition, environmental, social and demographic aspects, health factors and lifestyle contribute to the complexity of frailty ^41^ ^,^ ^45^.

Regarding the place of residence, living in a rural area had a direct negative effect on depressive symptoms, hemoglobin, and frailty; that is, residents of rural areas had a lower depression score, lower hemoglobin concentration, and a lower number of frailty components. Previous studies indicated a higher prevalence of frailty in rural areas, such as a cohort with older Chinese adults, with a significantly higher prevalence in rural areas (12% versus 5.3% in urban areas) ^46^. Another study conducted in South Korea found a prevalence of 17.4% in rural areas and 10.3% in urban areas ^47^. Xu et al. ^48^, in a review on the subject, highlight three main reasons for the higher prevalence in rural areas: lower socioeconomic status, limited accessibility to health services, and less healthy lifestyle in addition to limited awareness of health care.

However, other studies show a lower prevalence in rural areas, such as in England - in the *English Longitudinal Study of Ageing* (ELSA) cohort, a higher prevalence was observed in urban areas (7.3% versus 4.8% in rural areas) ^49^ - and in Brazil, where Pavarini et al. ^50^ reported a prevalence of 19.6% of frailty in older caregivers in urban areas versus 9.9% in rural areas. One possible explanation for this difference is that some of these studies showed better functional capacity of residents in rural areas ^50^, as they are more physically active when compared to residents in urban areas, which may also explain the lower frailty in this group. An additional hypothesis would be the migration of frail individuals to urban areas, seeking a better access to health services.

Regarding depression, the studies are not unanimous either, with results showing both higher and lower prevalence in rural areas. A meta-analysis including 18 studies from different regions of the world described a significantly higher prevalence of depression in urban areas in 10 studies, and only 3 studies showing a higher prevalence among rural residents (all Chinese studies) ^51^. In developed countries, the probabilities of depression were significantly higher among urban residents when compared to rural residents, but this association was not significant in developing countries ^51^. In a study with older caregivers from the countryside of the state of São Paulo, caregivers living in rural areas had better cognitive performance, less perceived stress and a higher level of hope ^50^. 

Regarding the negative effect of rural areas on hemoglobin, previous studies are controversial, sometimes conducted in the same country. For example, two studies conducted in Ecuador showed distinct results - a study in the city of Cuenca showed a higher prevalence of anemia among older adults living in rural areas ^52^ while Orces ^53^ described a higher prevalence in urban areas, analyzing data from the SABE Ecuador Study, arguing that the higher rate of population aging observed in urban areas when compared to rural areas may explain this difference. Another consideration that could help explain a higher urban prevalence is that air pollution may be directly associated with decreased hemoglobin levels ^54^. Our hypothesis is that the lower hemoglobin levels in rural areas are explained by worse sociodemographic conditions of residents, including greater food insecurity and worse access to health services.

Education had a positive direct path to cognition and a negative direct path to frailty, as well as an indirect negative path to frailty via hemoglobin, cognition, and depressive symptoms. This variable was added to the model for control purposes, since education is one of the main determinants of health known in the literature, and its relationship with all these outcomes is well established in both international and Brazilian studies ^55^ ^,^ ^56^ ^,^ ^57^ ^,^ ^58^ ^,^ ^59^.

We found a direct negative effect of the diet score on depressive symptoms and hemoglobin, and indirect paths to cognition and hemoglobin mediated by depressive symptoms. Inadequate diet may have a relationship with cognition and depressive symptoms. This study promotes a deeper discussion in two aspects: first, we used a score for four food groups (fruits/juice, vegetables, meat, and fish), surpassing isolated analyses and assessing dietary diversity. Second, we identified an indirect path from the lowest diet score to worse cognition via depressive symptoms, which was not significant in the direct paths, revealing an interrelationship between these conditions that has not been captured in traditional analyses. Although inadequate food intake is identified as one of the main risk factors for the syndrome, as it is directly related to malnutrition and weight loss ^3^ ^,^ ^60^. Our instrument was not able to measure protein and caloric adequacy, which may have weakened the relationship and failed to show a significant effect. 

In this sense, the number of teeth also had a direct positive effect on cognition, hemoglobin, and frailty, and indirect paths to cognition and frailty. Oral health is an important determinant of cognition in its various domains, as highlighted in a recent review ^61^. A 13-year cohort of older people in China (*Chinese Longitudinal Healthy Longevity Survey* - CLHLS) found that having more teeth was associated with better cognitive function. Also, the relationship between number of teeth and time was significant, as participants with more teeth showed a slower rate of cognitive decline than those with fewer teeth after assessing other covariates ^62^.

The relationship between number of teeth and frailty has also been assessed because malnutrition is one of the main risk factors for the syndrome. Another publication, with data from the *National Health and Nutrition Examination Surveys* (NHANES) 2011-2014, found that for each additional tooth, the relative risk of frailty was 0.99 (95%CI: 0.98-0.99) ^63^. A recent meta-analysis also revealed a negative association between the number of teeth and frailty, as older individuals with fewer than 20 teeth have a higher risk of frailty when compared to those with 20 or more teeth ^64^.

In this sense, the relationship between dentition and anemia could be explained by poor nutritional intake as well as a number of other mechanisms with “vicious circle” characteristics, such as diseases associated with both conditions, including cancer, HIV infection, bleeding gums, alcohol and tobacco abuse, among others. Anemia can also worsen the manifestations of oral diseases, as part of its clinical signs, including glossitis, recurrent canker sores, *Candida* infections, and angular cheilitis ^65^ ^,^ ^66^. Therefore, the relationship between these two conditions should be carefully analyzed and, if possible, using an individualized care plan.

Finally, the number of CNCDs was negatively associated with hemoglobin and positively associated with depressive symptoms and frailty and, although it did not show a direct path to cognition, it had an indirect effect via hemoglobin and depressive symptoms. Multimorbidity can be associated with depression through several mechanisms, including inflammaging and limitations imposed by the diseases ^67^. Then, the path to frailty was also expected - the authors of the frailty phenotype studied here claim that multimorbidity is a relevant stressor for the dysregulation of homeostasis, increasing the risk of frailty ^68^ ^,^ ^69^ ^,^ ^70^.

The reduction in hemoglobin in older people with CNCDs is also explained in the literature and can be attributed to distinct diseases, diagnosed or not, in addition to inflammation, hypogonadism, compromised hematopoiesis, among others ^71^ ^,^ ^72^. 

Regarding the significant negative indirect path of the number of diseases to better cognition, the literature seems inconsistent. Several studies have reported multimorbidity as a risk factor for cognitive impairment, mainly due to oxidative stress ^73^. However, other studies have reported that this relationship is mediated by confounding or causal factors. Aarts et al. ^74^ studied a normally aging adult population and found that simply counting the conditions was not significant. Data from NHANES 1999-2002 demonstrated that the association between multimorbidity and cognition lost significance when considering those individuals who observed the minimum recommendations of physical activity, which seemed to modulate the relationship ^75^. Our results are in line with this finding, since the relationship described here was modulated by depressive symptoms and hemoglobin.

These results should be interpreted considering some study limitations. This is a cross-sectional study in which “effect” and “path” indicate only statistical association, not causality. Important variables were not included in the model due to the impossibility of considering all the characteristics of the questionnaires. Lifestyle variables, such as smoking and alcoholism, were not considered due to the lack of consistent interactions, but they may be relevant in the history of frailty. The strengths of this study include the fact that it uses a unique and innovative model that was able to identify the interrelationship of four factors - cognition, hemoglobin, depression, and frailty - in a large sample, representative of older Brazilian adults, with methodological rigor in data collection and management.

In terms of public health, the fact that some factors can be associated with frailty both directly and mediated by other conditions is important to help understand the syndrome and its various facets, so that broader prevention and intervention measures can be proposed, considering the provision of basic living conditions, such as access to health care and education, maintenance of healthy habits such as dietary variety, management of CNCDs, and oral health care.
