# Supplementary material for: Interrelationships of frailty, hemoglobin, cognition, and depressive symptoms in aging: a path analysis of the ELSI-Brazil study
Source: Cad Saude Publica. 2025 Apr 28;41(3):e00105124. doi: 10.1590/0102-311XEN105124 (PMC12055011; doi:10.1590/0102-311XEN105124)
Supplement: Translation

# Translation: As inter-relações entre fragilidade, hemoglobina, cognição e sintomas depressivos no envelhecimento: uma análise de caminhos do estudo ELSI-Brasil

**DOI:** 10.1590/0102-311XPT105124

**Authors:** Ligiana Pires Corona, Gabriela Benatti de Oliveira, Lara Vilar Fernandes, Natalie Bitencourt Ramos, Carolina Neves Freiria, Luciana Scarlazzari Costa

**Affiliations:** 1 Faculdade de Ciências Aplicadas, Universidade Estadual de Campinas, Limeira, Brasil.; 2 Universidade São Francisco, Bragança Paulista, Brasil.

**Keywords:** Fragilidade, Anemia, Depressão, Modelagem de Equações Estruturais

## Abstract

Este estudo objetivou avaliar as inter-relações entre anemia, depressão e cognição, bem como alguns de seus fatores associados, para compreender os caminhos até a fragilidade. Foram utilizados dados de 2.174 participantes da linha de base do *Estudo Longitudinal de Saúde dos Idosos Brasileiros* (ELSI-Brasil). Utilizou-se análise de caminhos para avaliar as relações entre variáveis exógenas (local de residência, escolaridade, número de doenças, escore de alimentação e número de dentes naturais), uma endógena (fragilidade) e três mediadoras (cognição, depressão e nível de hemoglobina). Nas análises, cognição e hemoglobina tiveram caminho negativo para fragilidade, e sintomas depressivos, positivo. Entre as variáveis exógenas, zona rural teve efeito negativo sobre hemoglobina, sintomas depressivos e fragilidade; maior escolaridade apresentou caminho positivo para cognição; número de doenças teve caminho negativo para hemoglobina, e positivo para sintomas depressivos e fragilidade; escore de alimentação apresentou caminho negativo para hemoglobina e sintomas depressivos; número de dentes teve efeito positivo na cognição e hemoglobina, e negativo na fragilidade. Dois caminhos sem efeito direto tiveram efeitos indiretos significativos - zona rural apresentou relação indireta com cognição via hemoglobina e sintomas depressivos; e escolaridade teve caminho indireto para fragilidade, mediado pelas três variáveis intermediárias. Esses resultados mostram complexas inter-relações entre fragilidade, hemoglobina, cognição e sintomas depressivos, importantes para compreender a síndrome de maneira ampla, de modo que se possa planejar medidas mais abrangentes de prevenção e intervenção.

## Introdução

As síndromes geriátricas, como a fragilidade, preocupam cada vez mais os profissionais da saúde, devido ao envelhecimento populacional exponencial das últimas décadas. A prevalência de fragilidade varia de acordo com o critério diagnóstico utilizado ^1^. No Brasil, encontrou-se prevalência de 8,1% entre indivíduos de 60-69 anos e de 20,9% naqueles com 70 anos ou mais ^2^. É um fator que aumenta o risco de declínio funcional e cognitivo, hospitalização, morbidade e mortalidade, e, consequentemente, piora a qualidade de vida, apresenta menor longevidade e aumento com gastos em saúde.

A fisiopatologia da fragilidade caracteriza-se por um desequilíbrio na homeostase, criando um ciclo patológico em espiral negativa de declínio funcional, proposto por Fried et al. ^3^, manifestado clinicamente por redução de força, fadiga, lentidão, baixa atividade física e perda de peso, compreendido como fenômeno complexo e interconectado ^4^.

A fragilidade tem sido associada à anemia ^5^ ^,^ ^6^, à cognição ^7^ ^,^ ^8^, à depressão ^9^ ^,^ ^10^ ^,^ ^11^, à alimentação ^12^ ^,^ ^13^, ao estilo de vida ^14^ ^,^ ^15^, a doenças crônicas não transmissíveis (DCNT) ^16^ e à sarcopenia ^17^, porém, até o momento, há evidências limitadas sobre as complexas relações dessas condições até a manifestação clínica da fragilidade.

A compreensão dessa complexidade pode contribuir com o desenvolvimento de ações de prevenção e tratamento da fragilidade de maneira mais ampla, assim como servir de subsídio para a criação de políticas públicas visando a melhor qualidade de vida e longevidade da população. Diante disso, o objetivo deste estudo foi avaliar as inter-relações entre anemia, depressão, e cognição, bem como alguns de seus fatores associados, para avaliar os caminhos até a fragilidade.

## Métodos

Este estudo utiliza dados da linha de base do *Estudo Longitudinal de Saúde dos Idosos Brasileiros* (ELSI-Brasil), pesquisa de base domiciliar realizada em 2015-2016 com amostra nacional representativa da população com 50 anos ou mais. Detalhes metodológicos do estudo (seleção, amostra, procedimentos) estão descritos na página da internet do estudo: https://elsi.cpqrr.fiocruz.br/ e publicações anteriores ^18^.

A pesquisa foi conduzida em 70 municípios nas cinco grandes regiões do Brasil, com amostra final de 9.412 participantes. Uma subamostra foi selecionada também de maneira probabilística para coleta de sangue, totalizando 2.174 pessoas. As amostras sanguíneas foram coletadas nos domicílios, preparadas e enviadas para o laboratório central para análises. Para garantir a qualidade e viabilidade das amostras, obedeceu-se a um protocolo de melhores práticas, que incluiu embalagem com gelo seco e monitoramento da temperatura durante o traslado ^19^.

O ELSI-Brasil foi aprovado pelo Comitê de Ética em Pesquisa da Fundação Oswaldo Cruz, Minas Gerais (CAAE 34649814.3.0000.5091), e todos os entrevistados assinaram termo de consentimento livre e esclarecido (TCLE) para participação no estudo ^18^ ^,^ ^19^.

A principal variável desfecho é a fragilidade, definida como a somatória de cinco critérios apresentados em publicações prévias ^2^ ^,^ ^3^ ^,^ ^4^: autorrelato da perda de peso não intencional (últimos três meses), fraqueza (força de preensão palmar no quintil inferior estratificada por sexo e quartis de índice de massa corporal - IMC), baixa velocidade de marcha (quintil superior do tempo de caminhada de três metros, estratificado por sexo e altura), exaustão (respostas a duas questões do *Questionário de Depressão do Centro de Estudos Epidemiológicos* - CES-D, acrônimo em inglês) ^20^ ^,^ ^21^ e baixo nível de atividade física (menor quintil de gasto calórico semanal calculado a partir do questionário *Versão Abreviada do Questionário Internacional de Atividade Física* - IPAQ ^22^, acrônimo em inglês, estratificado por sexo).

Foram selecionadas três condições como possíveis mediadoras: níveis séricos de hemoglobina (mg/L), cognição (soma das memórias imediata e tardia, e fluência verbal; quanto maior, melhor) ^23^ ^,^ ^24^ e número de sintomas depressivos, identificados pela escala de depressão do CES-D (variando de 0 a 8, quanto maior o escore, maior o número de sintomas depressivos) ^21^.

As variáveis explicativas utilizadas foram: local de residência (área urbana, área rural); escolaridade (não estudou, primário, ginásio, colegial, superior); número de dentes naturais na boca (nenhum, 1-9, 10-19, ≥ 20); número de doenças (soma de 13 DCNT autorreferidas: hipertensão, diabetes, hipercolesterolemia, doenças cardíacas, acidente vascular cerebral, doenças pulmonares crônicas, artrite/reumatismo, osteoporose, problemas crônicos de coluna, câncer, insuficiência renal crônica, doenças de Parkinson ou Alzheimer); e grupos alimentares com consumo adequado (consumo de frutas/suco, verduras e carnes pelo menos cinco vezes na semana e consumo de peixe pelo menos uma vez na semana; soma variando de 0 a 4 grupos adequados).

Para as análises descritivas, utilizou-se média (erro padrão), valores mínimos e máximos e porcentagens. Para avaliar as inter-relações entre as variáveis e o escore de fragilidade, foram ajustados modelo de equações estruturais e análise de caminhos considerando cinco variáveis exógenas (local de residência, escolaridade, número de doenças, escore de alimentação e número de dentes naturais), uma endógena (fragilidade) e três mediadoras, podendo ser tanto exógenas quanto endógenas (cognição, depressão e nível de hemoglobina). Avaliou-se a adequação do modelo pelo teste qui-quadrado (erro médio quadrado de aproximação - RMSEA; valores < 0,05 indicam excelente ajuste), raiz quadrada média residual padronizada (SRMR; aceitáveis valores < 0,08), índice de Tucker-Lewis (TLI) e índice de ajuste comparativo (CFI) variam de 0 a 1; quanto mais próximo de 1, melhor o ajuste.

Os dados foram analisados utilizando o software Stata, versão 14.0 (https://www.stata.com), com nível de 5% de significância, utilizando pesos amostrais calculados especificamente para os que forneceram amostra sanguínea por meio do pacote *survey* para amostra complexa.

## Resultados

A maior parte dos participantes do estudo ELSI-Brasil mora na área urbana e estudou até o primeiro grau (59,8%); apresenta, em média, 2,38 doenças; tem nível médio de hemoglobina de 13,71mg/dL e 1,06 componentes de fragilidade (dados não apresentados em tabela).

Os efeitos diretos entre as variáveis estão apresentados na Figura 1. Cognição e hemoglobina apresentaram caminho negativo para fragilidade, e sintomas depressivos, positivo. Entre as variáveis exógenas, zona rural apresentou efeito negativo sobre hemoglobina, sintomas depressivos e fragilidade; maior escolaridade apresentou caminho positivo para cognição; número de doenças apresentou caminho negativo para hemoglobina, e positivo para sintomas depressivos e fragilidade; escore de alimentação apresentou caminho negativo para hemoglobina e sintomas depressivos; e número de dentes teve efeito positivo na cognição e hemoglobina, e negativo na fragilidade.

**Figura 1:** Modelo das relações diretas entre variáveis na análise de caminhos.

Nota: apenas as linhas inteiriças representam associações significativas (p < 0,05). Categorias de referência das variáveis qualitativas - idade (50-59 anos); local de residência (urbano); escolaridade (não estudou); número de dentes naturais na boca (nenhum).

A Tabela 1 contempla os efeitos totais da análise de caminhos, e a Tabela 2 apresenta os dados de efeitos indiretos observados no modelo.

**Tabela 1:** Efeitos totais da análise de caminhos.

Efeitos totais	Coeficiente padronizado	Erro padrão	Valor de p
Cognição			
Sintomas depressivos	-0,006	0,008	0,016
Nível sérico de hemoglobina	0,109	0,105	< 0,001
Local de residência	-0,012	0,394	0,528
Escolaridade	0,437	0,131	< 0,001
Número de doenças	-0,030	0,085	0,113
Escore alimentação	0,009	0,139	0,609
Número de dentes naturais	0,143	0,130	< 0,001
Sintomas depressivos			
Local de residência	-0,064	0,153	0,003
Escolaridade	-0,034	0,051	0,158
Número de doenças	0,187	0,033	< 0,001
Escore alimentação	-0,102	0,054	< 0,001
Número de dentes naturais	-0,005	0,050	0,819
Hemoglobina			
Sintomas depressivos	-0,059	0,012	0,008
Local de residência	-0,079	0,083	< 0,001
Escolaridade	0,024	0,028	0,318
Número de doenças	-0,124	0,018	< 0,001
Escore alimentação	-0,076	0,029	0,001
Número de dentes naturais	0,105	0,028	< 0,001
Fragilidade			
Cognição	-0,320	0,003	< 0,001
Sintomas depressivos	0,154	0,009	< 0,001
Nível sérico de hemoglobina	-0,099	0,017	< 0,001
Local de residência	-0,046	0,064	0,034
Escolaridade	-0,170	0,021	< 0,001
Número de doenças	0,182	0,014	< 0,001
Escore alimentação	-0,014	0,022	0,512
Número de dentes naturais	-0,112	0,021	< 0,001

**Tabela 2:** Efeitos indiretos da análise de caminhos.

Efeitos indiretos	Mediador	Coeficiente padronizado	Erro padrão	Valor de p
Cognição				
Sintomas depressivos	Nível sérico de hemoglobina	-0,006	0,008	0,016
Local de residência	Nível sérico de hemoglobina/Sintomas depressivos	-0,008	0,058	0,002
Escolaridade	Nível sérico de hemoglobina/Sintomas depressivos	0,003	0,017	0,325
Número de doenças	Nível sérico de hemoglobina/Sintomas depressivos	-0,013	0,015	< 0,001
Escore alimentação	Nível sérico de hemoglobina/Sintomas depressivos	-0,008	0,020	0,004
Número de dentes naturais	Nível sérico de hemoglobina/Sintomas depressivos	0,011	0,020	0,001
Nível sérico de hemoglobina				
Local de residência	Sintomas depressivos	0,004	0,007	0,049
Escolaridade	Sintomas depressivos	0,002	0,002	0,213
Número de doenças	Sintomas depressivos	-0,011	0,004	0,011
Escore alimentação	Sintomas depressivos	0,006	0,003	0,021
Número de dentes naturais	Sintomas depressivos	-0,000	0,001	0,819
Fragilidade				
Sintomas depressivos	Nível sérico de hemoglobina	0,006	0,001	0,021
Nível sérico de hemoglobina	Cognição	-0,035	0,005	< 0,001
Local de residência	Nível sérico de hemoglobina/Sintomas depressivos/Cognição	-0,000	0,023	0,950
Escolaridade	Nível sérico de hemoglobina/Sintomas depressivos/Cognição	-0,147	0,011	< 0,001
Número de doenças	Nível sérico de hemoglobina/Sintomas depressivos/Cognição	0,046	0,006	< 0,001
Escore alimentação	Nível sérico de hemoglobina/Sintomas depressivos/Cognição	-0,013	0,008	0,089
Número de dentes naturais	Nível sérico de hemoglobina/Sintomas depressivos/Cognição	-0,053	0,008	< 0,001

Medidas de adequação do ajuste pela análise de caminhos: CFI (índice de ajuste comparativo) = 0,999; TLI (índice de Tucker-Lewis) = 0,970; SRMR (raiz quadrada média residual padronizada) = 0,005; RMSEA (erro médio quadrado de aproximação) = 0,027; teste qui-quadrado para qualidade de ajuste = 0,000; razão qui-quadrado (χ^2^/g.l.) = 0,114. O R^2^ total do modelo foi de 35%.

Dois caminhos sem efeito direto tiveram efeitos indiretos significativos - viver na zona rural apresentou relação indireta com cognição via hemoglobina e sintomas depressivos; e escolaridade apresentou caminho indireto para fragilidade, mediado pelas três variáveis intermediárias. O modelo mostrou-se adequado com relação aos parâmetros de ajuste.

## Discussão

Neste estudo, evidenciamos inter-relações entre fragilidade, hemoglobina, cognição e sintomas depressivos, e caminhos diretos e indiretos que alguns de seus determinantes clássicos apresentam. Até onde sabemos, este é o primeiro estudo que mostra todos esses efeitos em uma análise única com amostra representativa da população de 50 anos ou mais do país.

A fragilidade é uma síndrome geriátrica causada por mecanismos fisiopatológicos que desregulam múltiplos sistemas, esgotando reservas e comprometendo a homeostase diante de estressores. Envolve sistemas de produção, distribuição e uso de energia, incluindo processos hormonais, imunológicos, inflamatórios e neurológicos. Assim, o ciclo de redução de oferta e demanda energética leva a uma função comprometida intra e entre sistemas. Nesse cenário, a anemia está envolvida na fisiopatologia da síndrome, pela redução da capacidade de transporte de oxigênio, que pode levar à hipóxia tecidual ^25^ e, consequentemente, sarcopenia, osteoporose, disfunção cardíaca e progressão de doenças renais ^26^ ^,^ ^27^. Ainda, a anemia pode contribuir para as manifestações clínicas de fraqueza e intolerância ao exercício ^28^, componentes da fragilidade.

No entanto, a maioria dos trabalhos demonstra o efeito direto da anemia já instalada na síndrome de fragilidade, e não em gradiente de hemoglobina, além de serem estudos com características mais locais. No Brasil, o Estudo SABE (*Estudo Saúde, Bem-Estar e Envelhecimento*) mostrou essa relação em pessoas idosas do Município de São Paulo somente - níveis mais baixos de hemoglobina foram associados ao maior número de critérios positivos para fragilidade ^6^.

Menores níveis de hemoglobina também impactam na menor capacidade cognitiva e maior chance de transtornos depressivos - isso porque a hipóxia tecidual tem efeito importante no sistema nervoso, com duas possíveis direções de associação, de causa ou consequência.

A anemia pode levar à depressão por deficiência de vitaminas como folato e vitamina B12, que diminuem a produção de S-adenosilmetionina, ou pode aumentar a produção de homocisteína. Ao mesmo tempo, a fadiga e a falta de interesse para atividades de vida diária (como fazer compras e cozinhar), comuns em indivíduos depressivos, podem afetar a qualidade da alimentação, facilitando o desenvolvimento de anemia; o apetite também pode ser severamente impactado ^29^ ^,^ ^30^ ^,^ ^31^ ^,^ ^32^. Uma coorte de chineses demonstrou que a incidência de anemia em quatro anos de acompanhamento foi maior em indivíduos com sintomas depressivos, e quanto maior a pontuação no teste, maior a incidência de anemia ^33^. Neste estudo, quanto maior o número de sintomas depressivos, menor foi a concentração de hemoglobina circulante, mas não foi possível avaliar associações causais considerando a natureza transversal dos dados.

Quanto à capacidade cognitiva, o mesmo pode ocorrer - indivíduos com comprometimento cognitivo instalado podem ter dificuldade de compra e manipulação de alimentos, alimentar-se sozinhos, pior deglutição e, portanto, alimentação deficiente, podendo desencadear anemias carenciais ^34^. No entanto, a anemia como causa do comprometimento cognitivo é a via mais aceita. Duas revisões recentes mostraram que a anemia aumentou a incidência tanto de comprometimento cognitivo geral como demência, possivelmente pela hipóxia tecidual prolongada no tecido cerebral ^35^ ^,^ ^36^. A depressão também se associa positivamente à fragilidade física e cognitiva ^37^ ^,^ ^38^ por diversos mecanismos fisiológicos, com alterações na função sináptica, no transporte de proteínas e na função mitocondrial.

Essa inter-relação entre as várias condições exploradas neste trabalho (fragilidade, baixa hemoglobina, depressão e cognição) parece ter em comum o *inflammaging* - estado pró-inflamatório relacionado ao envelhecimento -, que aumenta o estresse oxidativo, impactando negativamente os sistemas envolvidos ^39^ ^,^ ^40^. A inflamação pode estar associada diretamente à fragilidade por mecanismos independentes ^41^, bem como à alteração na resposta eritropoiética ^42^, sendo aceita como determinante no desenvolvimento de anemia crônica em pessoas idosas ^27^ ^,^ ^42^ ^,^ ^43^. Segundo Guralnik et al. ^44^, pelo menos um terço das anemias no envelhecimento pode ser atribuído às DCNT (em especial, as renais) e/ou à inflamação.

Além disso, aspectos ambientais, sociais e demográficos, fatores de saúde e estilo de vida contribuem para a complexidade do quadro da fragilidade ^41^ ^,^ ^45^.

Em relação à área, residir na zona rural teve efeito negativo direto para sintomas depressivos, hemoglobina e fragilidade, ou seja, moradores da área rural apresentaram menor escore de depressão, menor concentração hemoglobina e menor número de componentes de fragilidade. Estudos anteriores já indicaram prevalência maior de fragilidade na área rural, como uma coorte com pessoas idosas chinesas, com prevalência significativamente maior nas áreas rurais (12%, *versus* 5,3% áreas urbanas) ^46^. Outro estudo da Coreia do Sul mostrou prevalência de 17,4% na área rural e 10,3% na urbana ^47^. Xu et al. ^48^, em revisão sobre o tema, levantam três principais razões para maior prevalência na zona rural: menor nível socioeconômico; acessibilidade limitada a serviços de saúde; e estilo de vida menos saudável, além de consciência limitada dos cuidados de saúde.

No entanto, outros estudos apontaram menor prevalência em áreas rurais, como na Inglaterra - na coorte do *Estudo Longitudinal do Envelhecimento Inglês* [*English Longitudinal Study of Ageing*] (ELSA, acrônimo em inglês), houve maior prevalência na área urbana (7,3%, *versus* 4,8% na área rural) ^49^ -, e no Brasil, onde Pavarini et al. ^50^ relataram prevalência de 19,6% de fragilidade em pessoas idosas cuidadoras da área urbana, *versus* 9,9% na área rural. Uma possível explicação para essa diferença é que alguns desses estudos mostraram melhor capacidade funcional dos moradores das áreas rurais ^50^, por serem mais ativos fisicamente quando comparado aos do meio urbano, o que também pode explicar a menor fragilidade neste grupo. Uma hipótese adicional seria a da migração das pessoas mais frágeis para as zonas urbanas, buscando maior acesso a serviços de saúde.

Em relação à depressão, as pesquisas também não são unânimes, com resultados tanto de maior quanto de menor prevalência nas áreas rurais. Uma metanálise incluindo 18 estudos de várias regiões do mundo descreveu prevalência de depressão significativamente maior em meios urbanos em dez estudos, e maior entre residentes rurais em três estudos (todos chineses) ^51^. Nos países desenvolvidos, as probabilidades de depressão foram significativamente maiores entre residentes urbanos do que rurais, mas essa associação não foi significativa nos países em desenvolvimento ^51^. Na pesquisa com idosos cuidadores do interior de São Paulo, os de áreas rurais tiveram melhor desempenho cognitivo, menos estresse percebido e maior nível de esperança ^50^.

Quanto ao efeito negativo da zona rural na hemoglobina, estudos prévios são controversos, por vezes até no mesmo país. Dois estudos realizados no Equador, por exemplo, mostraram resultados contrastantes - um estudo da cidade de Cuenca mostrou maior prevalência de anemia entre pessoas idosas da zona rural ^52^; já Orces ^53^ descreveu maior prevalência na zona urbana, analisando dados do Estudo SABE Equador, discutindo que a maior taxa de envelhecimento populacional observada no meio urbano em relação à zona rural pode explicar essa diferença. Outra consideração que poderia ajudar a explicar uma maior prevalência urbana é que a poluição do ar pode ser diretamente associada a piores níveis de hemoglobina ^54^. Nossa hipótese é que os menores níveis de hemoglobina na zona rural sejam explicados pelas piores condições sociodemográficas dos moradores, refletindo maior insegurança alimentar e pior acesso à saúde.

A escolaridade teve caminho direto positivo para cognição e negativo para fragilidade, e caminho negativo indireto para fragilidade via hemoglobina, cognição e sintomas depressivos. Essa variável foi adicionada ao modelo visando controle, já que escolaridade é um dos principais determinantes de saúde conhecidos na literatura, e sua relação com todos esses desfechos é bem estabelecida tanto em estudos internacionais como brasileiros ^55^ ^,^ ^56^ ^,^ ^57^ ^,^ ^58^ ^,^ ^59^.

Encontramos efeito negativo direto do escore de alimentação nos sintomas depressivos e hemoglobina, e caminhos indiretos para cognição e hemoglobina, mediados por sintomas depressivos. Alimentação inadequada pode ter relação dupla com cognição e sintomas depressivos. Este estudo avança a discussão por dois fatores: primeiro, usamos um escore para quatro grupos alimentares (frutas/suco, verduras, carnes e peixe), superando análises isoladas e avaliando a diversidade alimentar. Segundo, identificamos um caminho indireto do menor escore de alimentação para pior cognição via sintomas depressivos, não significativo nos caminhos diretos, revelando inter-relação entre essas condições não captada em análises tradicionais. Apesar de a ingestão alimentar inadequada ser apontada como um dos principais fatores de risco para a síndrome, já que está diretamente relacionada à desnutrição e perda de peso ^3^ ^,^ ^60^, nosso instrumento não é capaz de medir adequação proteica e calórica, o que pode ter enfraquecido a relação e não apresentado efeito significativo.

Nesse sentido, o número de dentes também teve efeito positivo direto para cognição, hemoglobina e fragilidade, bem como caminhos indiretos para cognição e fragilidade. A saúde oral é um importante determinante da cognição em seus diversos domínios, como apontado em revisão recente ^61^. Uma coorte de 13 anos com pessoas mais velhas na China (*Pesquisa Longitudinal Chinesa sobre Longevidade Saudável* - CLHLS, acrônimo em inglês) destacou que ter mais dentes associou-se a uma melhor função cognitiva; ademais, a interação entre número de dentes e tempo foi significativa - participantes com mais dentes apresentaram ritmo mais lento de declínio cognitivo do que aqueles com menos dentes após controlar outras covariáveis ^62^.

Também já foi explorada a relação de dentição e fragilidade, novamente, pela má nutrição ser um dos principais fatores de risco para a síndrome. Outra publicação, com dados do Pesquisa Nacional de Exame de Saúde e Nutrição (NHANES, acrônimo em inglês), dos Estados Unidos, no período de 2011-2014, relatou que para cada dente adicional, o risco relativo de fragilidade foi de 0,99 (IC95%: 0,98-0,99) ^63^. Uma metanálise recente também revelou associação negativa entre número de dentes e fragilidade - idosos com menos de 20 dentes enfrentam risco maior de fragilidade em comparação aos com 20 dentes ou mais ^64^.

Ainda nesse sentido, a relação entre dentição e anemia poderia ser explicada pela ingestão nutricional deficiente, mas também por uma série de outros mecanismos, muitos deles com características de “ciclo vicioso”, como doenças associadas a ambas condições, como câncer, infecção por HIV, sangramento gengival, abuso de álcool e tabaco, entre outras; a anemia também pode piorar as manifestações de doenças orais, como parte de seus sinais clínicos, como glossite, aftas recorrentes, infecções por *Candida* e estomatite angular ^65^ ^,^ ^66^. Dessa forma, a análise da relação entre essas duas condições deve ser feita de maneira cuidadosa e, se possível, em um plano de cuidado individualizado.

Finalmente, o número de DCNT foi negativamente associado à hemoglobina, e positivamente aos sintomas depressivos e fragilidade, e, apesar de não mostrar caminho direto para cognição, teve efeito indireto via hemoglobina e sintomas depressivos. A multimorbidade pode ser associada à depressão por inúmeros mecanismos, desde *inflammaging* até pelas limitações impostas pelas doenças ^67^. Assim, também era esperado o caminho para fragilidade - os autores do fenótipo de fragilidade aqui estudado afirmam que a multimorbidade é um estressor relevante para a desregulação da homeostase, aumentando o risco de fragilidade ^68^ ^,^ ^69^ ^,^ ^70^.

A redução da hemoglobina em pessoas idosas com DCNT também é explicada na literatura, podendo ser atribuída a doenças diversas, diagnosticadas ou não, além de inflamação, hipogonadismo, hematopoiese comprometida, entre outros fatores ^71^ ^,^ ^72^.

Em relação ao caminho indireto negativo significativo do número de doenças para melhor cognição, a literatura parece inconsistente. Inúmeros trabalhos mostraram a multimorbidade como fator de risco para declínio cognitivo, principalmente devido ao estresse oxidativo ^73^. No entanto, outros estudos relataram que essa relação é mediada por fatores de confusão ou de linha causal. Aarts et al. ^74^ estudaram uma população adulta com envelhecimento normal e concluíram que a simples contagem das condições não foi significativa. Dados do NHANES 1999-2002 demonstraram que a associação entre multimorbidade e cognição perdeu significância quando se considerava aqueles que atingiam as recomendações mínimas de prática de atividade física, que parecia modular a relação ^75^. Assim, nossos resultados vão nesse sentido, pois a relação aqui descrita foi modulada pelos sintomas depressivos e hemoglobina.

Esses resultados devem ser interpretados considerando limitações. É um estudo transversal; “efeito” e “caminho” indicam apenas uma associação estatística, não causalidade. Variáveis importantes não foram incluídas no modelo devido à inviabilidade de considerar todas as características dos questionários. Variáveis de estilo de vida, como fumo e alcoolismo, não foram consideradas devido à falta de interações consistentes, mas podem ser relevantes na história da fragilidade. Entre os pontos fortes do estudo, trata-se de um modelo único e inovador que conseguiu identificar a inter-relação de quatro fatores - cognição, hemoglobina, depressão e fragilidade - em uma grande amostra, representativa de adultos mais velhos brasileiros, com rigor metodológico da coleta e manejo dos dados.

Em termos de saúde coletiva, a observação de que alguns fatores podem ser associados à fragilidade tanto diretamente como mediados por outras condições é importante para compreender a síndrome e suas diversas facetas, de modo que se possa propor medidas mais amplas de prevenção e intervenção, considerando o fornecimento de condições básicas de vida, como acesso à saúde e educação, manutenção de hábitos saudáveis como variedade alimentar, controle das DCNT e cuidados com a saúde bucal.
